# Strengthening the Evidence for a Causal Link between Reproductive Traits and Cognitive Function: Insights from Two‐Sample and Multivariable Mendelian Randomization

**DOI:** 10.1002/alz70861_108741

**Published:** 2025-12-23

**Authors:** Yujue Wang, Xia Wang, Yunyun Guo

**Affiliations:** ^1^ The Department of Gynecology at the Affiliated Hospital of Southwest Medical University, Luzhou, Sichuan China; ^2^ The Department of Obstetrics at the Affiliated Hospital of Southwest Medical University, Luzhou, Sichuan China; ^3^ The Ageing Epidemiology (AGE) Research Unit, School of Public Health, Imperial College London, London UK

## Abstract

**Background:**

Previous studies have suggested a link between reproductive traits and cognitive function, but evidence remains limited, and establishing causality is challenging. This study aims to assess the causal effects of reproductive traits—including age at first sexual intercourse (AFS), age at menarche (AAM), age at menopause (AAMo), age at first birth (AFB), birth weight of the first child, use of oral contraceptives (OCP), hormone replacement therapy (HRT), medical abortion, and number of live births—on seven cognitive‐related outcomes using Mendelian randomization (MR).

**Method:**

We performed MR analyses using genome‐wide association study (GWAS) data. GWAS summary statistics for reproductive traits were obtained from the ReproGen Consortium, UK Biobank, 23andMe, the Human Reproductive Behaviour Consortium, and FinnGen. Cognitive outcome data were drawn from 71 quality‐controlled and specialized cohorts, including the Lothian and Helsinki Birth Cohorts. The inverse variance weighted (IVW) method was used as the primary approach, with weighted median and MR Egger as complementary methods. Sensitivity analyses included Cochran's Q test, Egger intercept, and leave‐one‐out analysis. Multivariable MR (MVMR) was conducted to assess direct causal effects, adjusting for body mass index (BMI) and educational attainment.

**Result:**

Earlier AFB was associated with poorer cognitive performance (β = 0.116, 95% CI: 0.087 to 0.146, *p* < 0.001), lower fluid intelligence (β = 0.248, 95% CI: 0.186 to 0.309, *p* < 0.001), and weaker memory (β = 0.042, 95% CI: 0.012 to 0.071, *p* = 0.006). A higher number of live births was linked to reduced cognitive performance (β = –0.207, *p* = 0.048) and fluid intelligence (β = –0.654, *p* < 0.001). HRT use was associated with slower performance in Pairs Matching and lower scores in Symbol Digit Substitution tests. These effects remained after adjusting for BMI and education.

**Conclusion:**

This MR study supports a causal relationship between reproductive traits and cognitive outcomes. Further large‐scale prospective studies are warranted for confirmation.

